# The Correlation of the IETA Ultrasound Score with the Histopathology Results for Women with Abnormal Bleeding in Western Romania

**DOI:** 10.3390/diagnostics11081342

**Published:** 2021-07-26

**Authors:** Alexandru Marius Furau, Mirela Marioara Toma, Cringu Ionescu, Cristian Furau, Simona Bungau, Mihai Dimitriu, Delia Mirela Tit, Gheorghe Furau, Izabella Petre, Marius Craina

**Affiliations:** 1 Department of Oncology, “Vasile Goldis” Western University of Arad, 310414 Arad, Romania; marius.furau@yahoo.com; 2 Doctoral School of Medicine, “Victor Babes” University of Medicine and Pharmacy, 300041 Timisoara, Romania; 3 Department of Pharmacy, Faculty of Medicine and Pharmacy, University of Oradea, 410028 Oradea, Romania; mire.toma@yahoo.com; 4 Doctoral School of Biological and Biomedical Sciences, University of Oradea, 410087 Oradea, Romania; 5 Clinical Department 13, Faculty of Medicine, “Carol Davila” University of Medicine and Pharmacy, 030167 Bucharest, Romania; antoniuginec@yahoo.com (C.I.); drmihaidimitriu@yahoo.com (M.D.); 6 Department of Pathophysiology, Faculty of Medicine, “Vasile Goldis” Western University of Arad, 310414 Arad, Romania; cristianfurau@gmail.com; 7 Department of Obstetrics and Gynecology, Emergency Clinical County Hospital of Arad, 310037 Arad, Romania; gfurau@yahoo.com; 8 Department of Obstetrics and Gynecology, “Vasile Goldis” Western University of Arad, 310414 Arad, Romania; 9 Department of Obstetrics and Gynecology, Faculty of Medicine and Pharmacy, “Victor Babes” University of Medicine and Pharmacy, 300041 Timisoara, Romania; dr.petreizabella@yahoo.com (I.P.); mariuscraina@hotmail.com (M.C.)

**Keywords:** endometrial cancer, endometrial hyperplasia, IETA scores, histology, uterine bleeding

## Abstract

In the early differential diagnosis of endometrial cancer (EC), decisive and mandatory histological aspects are considered, in addition to obvious clinical manifestations. In addition, sonographic aspects are characteristic in relation to the stage, degree, and histological types of identified cancer. This bi-center retrospective observational study included 594 women with abnormal uterine bleeding outside pregnancy, for which a biopsy was performed in the Obstetrics and Gynecology Departments of the Emergency County Hospitals of Arad and Timis Counties, Romania, between 2015 and 2019. Most of the cases were represented by EC or endometrial hyperplasia (EH). Of the 594 cases, 25.5% (*n* = 153) were EC at women aged between 41 and 85 years. High International Endometrial Tumor Analysis (IETA) scores (3, 4) were associated with a relative risk of 2.9335 compared with other endometrial lesions (95% CI 2.3046 to 3.734, *p* < 0.0001, NNT 1.805). Histological aspects and pelvic ultrasound using IETA scores represent valuable noninvasive assets in diagnosing and differentiating endometrial cancer from benign uterine pathology.

## 1. Introduction

Endometrial cancer (EC) is the sixth most diagnosed cancer in women [1], being easily recognizable, especially in the form of the three major histopathological types (EEC endometrium, serous, and clear cell), estrogen-dependent type I and estrogen-independent type II sharing many common etiologic factors [2]. EC has a distinct natural history and genetic etiology and is associated with obvious clinical manifestations. The incidence of EC increases with age and is most common between 45 and 65 years [2]. The staging system of the International Federation of Gynecology and Obstetrics (FIGO) was updated mainly for EC, and their immunophenotypic variants have a lot of implications for differential diagnosis, evolution, prognosis, and therapeutic approach [3].

In general, endometrioid adenocarcinoma represents 80% of all EC, displaying low grade, frequent in nulliparous and in women with Body Mass Index (BMI) over 25. Villoglandular (papillary) endometrioid adenocarcinoma is also a relatively common type. Endometrioid adenocarcinoma with secretory differentiation, endometrial serous carcinoma, clear-cell endometrioid adenocarcinoma, squamous cell carcinoma, mixed cell endometrial carcinoma, mucinous adenocarcinoma, undifferentiated endometrial carcinoma, pavement-cell carcinoma and carcinosarcoma of the uterus are all rare but aggressive, some of them, such as serous endometrioid adenocarcinoma not being hormone-sensitive [4].

Considering the pathophysiologic perspective, EC have the following two types:

- Type 1, which includes endometrioid and mucinous carcinoma (usually corelated with long term enhanced levels of estrogen, thus conducting to persistent proliferative stimulation of the endometrium); *PTEN, KRAS,* and *PAX2* gene alterations are common as well as atypical endometrial hyperplasia/endometrioid intraepithelial neoplasia, which is regarded as the precursor lesion).
- Type 2, which includes serous, clear cell, undifferentiated carcinoma and carcinosarcoma, being tumors that have a lesser correlation with unopposed estrogen exposure) [3,5].

In term of sonographic features, different endometrial pathologies are characterized by various ultrasound (US) features, respectively different mathematical models that use Doppler US in shades of gray or colored (i.e., endometrial echogenicity, vessel morphology, or color content of endometrial scanning); they were designed to facilitate the calculation of the risk of malignancy in women with postmenopausal bleeding and sonographic endometrium (thickness ≥ 4.5 mm), but without fluid accumulated in the uterine cavity [6,7,8]. The RSNA considers that in healthy postmenopausal subjects, the endometrium generally measures ≤5 mm [9]. If there is a perimenopausal hyperplasia, it may return to normal, sometimes spontaneously, a few months after menstruation stops. If it does not return to normal, the endometrium may continue to thicken and develop as complex hyperplasia or complex hyperplasia with atypia, resulting in endometrial cancer. [7,9].

EC displays a heterogeneous characteristic, having a very complex molecular pathogenesis. It also requires diagnostic methods such as immunohistochemistry and molecular testing including differentiation of endocervical and endometrial primaries by curettage species, subtyping of high-quality endometrial cancers into biologically significant categories, assessment of patients for Lynch syndrome, and identification of those who may benefit from specific/targeted therapies [10,11]. Apart of these, using the International Endometrial Tumor Analysis (IETA) terminology, clinicians must describe the sonographic EC characteristics, correlated to grade, stage, and histological type [9,10].

This retrospective representative bi-center study performed in Western Romania analyzes over a significant period (5 years) the clinical and diagnostic correlations between histopathological aspects encountered in abnormal uterine bleeding, especially in endometrial cancer, and the role that an ultrasonographic score (such as IETA score) may have in the management of these cases. The results offer valuable information for clinicians, in optimizing the management of abnormal uterine bleeding, in the positive and differential diagnosis of endometrial cancer. The relevance of the data obtained is given by the long term of the study and by the number of the investigated patients. Additionally, as far as we know, this study is the only one on this topic performed in Romania, so it provides valuable data for the southern-east part of Europe.

## 2. Materials and Methods

### 2.1. Study Design

**Figure 1:**
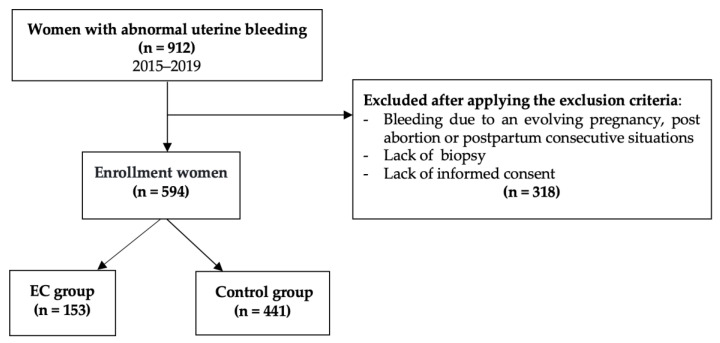
Flow chart describing the selection criteria of the patients.

A bi-center observational retrospective study was designed; data were collected from the archive of the two most representative hospitals for each of the county (Emergency Clinical County Hospitals of Arad and Timis, Arad, Romania) and they were analyzed in a retrospective case-control, descriptive, and analytical way. A five-year period (2015–2019) was considered, during which 594 cases of women with abnormal uterine bleeding (AUB) were hospitalized/registered, biopsy being performed in each case. The following criteria have led to the exclusion of some subjects: Bleeding due to an evolving pregnancy and post abortion/postpartum consecutive situations, Figure 1 presents the flow chart describing the enrolment criteria for the patients. 

Two groups were defined from the perspective of malignancy: the case group was represented by EC (*n* = 153) and the control group by EH and other gynecological pathologies, which presented genital bleeding for which a biopsy was taken (*n* = 441). 

The following aspects were studied:

- Clinical and histopathological findings;
- The presence of common histopathological associations and its relevance, if it exists;
- Common features for each of the identified cohorts: EC, EH, other lesions;
- The correlations between IETA ultrasonographical scores and the histopathological findings.

The study was approved by Ethical Committees of the University of Medicine and Pharmacy Victor Babes Timisoara (23/03.04.2020).

### 2.2. Endometrial Cancers Characterization

The diagnosis of EC was established on the histopathological examination of diagnostic biopsies or tissues taken during surgical procedures. The biopsied tissue samples were fixed with formalin and embedded in paraffin. The resulting sections were stained with hematoxylin-eosin and an Optic Zeus Primo Star Microscope was used for analysis. The type and grading of the tumors were made using the WHO classification for this pathology [12]. 

### 2.3. IETA Characteristics in Endometrial Pathology

**Figure 2:**
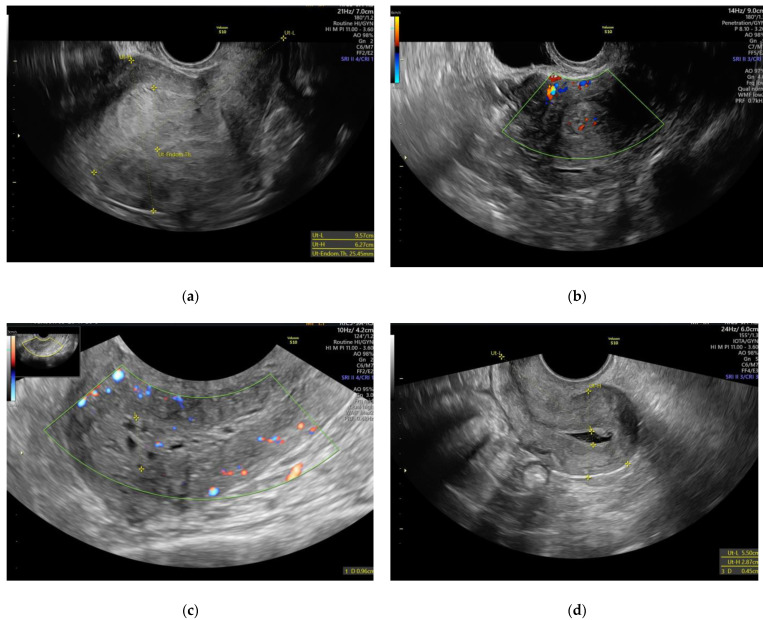

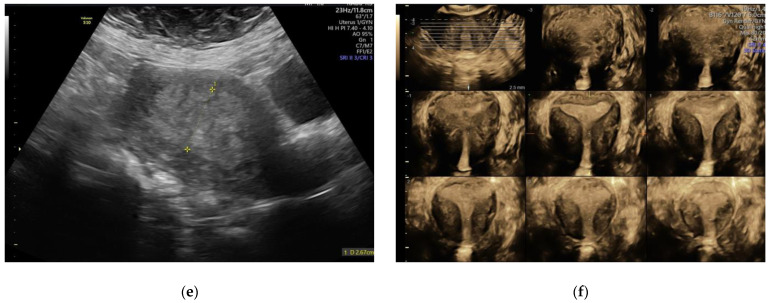
Ultrasonographic images. (**a**) EH—the endometrial line is not clearly defined, discreet hyperechogenic aspect of the endometrium (endometrial thickness = 25.45 mm); (**b**) EH—2D Color Doppler. Dispersed endometrial vessels and irregular endo-myometrial junction can be observed; (**c**) 2D Color Doppler—dispersed endometrial vessels, hyperechogenic endometrium without an endometrial line can be seen; (**d**) echogenic aspect of the endometrial fluid. A lesion can be observed (polyp), with an implant base of less than 25% of the endometrial surface; (**e**) endometrial adenocarcinoma. Irregular and infiltrative aspect of the endometrium especially in the endo-myometrial junction, endometrial thickness of 26.7 mm; (**f**) 3D TUI (Tomographic Ultrasound Imaging) Image acquisition.

All scanned patients were placed in lithotomy position after emptying the urinary bladder. The uterus was examined in a sagittal section from one horn to the other and in a transverse section from the cervix to the uterine fundus. The presence of the following findings was noted: adenoma, polyps, leiomyoma, etc. The antero-posterior diameter and the endometrial thickness were measured using a sagittal section. After visualizing the entire uterus, the image was zoomed in on the uterine corpus. The obtained image was enhanced for a morphological sonographic description using a grey scale, for the vascularization color, Doppler was used according to the IETA definitions and recommendations [5]. Complete description according to these criteria was available for 420 patients, for which based on the IETA algorithm the following were performed: Echogenity of the endometrium, description of the endometrial-myometrial junction, vascular aspect, intrauterine fluid, and IETA score [5]. All US examinations of the patients were performed by physicians (medical doctors) and gynecologists, with advanced competencies and extensive experience in ultrasonography in obstetrics and gynecology and using high-end ultrasound equipment (Voluson S10 Expert and Voluson E8 Expert, both from GE Ultrasound Korea, Ltd., Seongnam-Si Gyeonggi, Korea). Figure 2 shows some of the ultrasonographic images obtained during this study.

### 2.4. Statistical Analysis

The following were performed to validate the quality of the obtained data: sensibility, specificity, positive, and negative predictive value PPV, NPV, as well as prevalence rate, relevant ratio, and percentages. For continuous types of numerical data, we used the mean and confidence interval (CI) of 95%, while for category type variables we used the value and percentage. The significant statistical value was considered *p* ≤ 0.05.

## 3. Results

### 3.1. Demographic, Clinical, and Histopathological Results

The mean age of the patients included in the study was 56 years. Most of them were postmenopausal (75.1%). Demographic data and clinical status are presented in Table 1.

Of the 594 cases, 25.5% (*n* = 153) were EC at women aged between 41 and 85 years. Average age at diagnosis was 64.14 (SD 8.794) for EC and lower for non-oncogenic pathology with extreme values of 58.33 (SD13.172) for cervicitis and 49.70 (10.279) for typical EH. The most frequent histopathological type of EC was represented by endometrioid adenocarcinoma (*n* = 101), followed by villoglandular endometrioid adenocarcinoma (*n* = 10) and squamous endometrioid adenocarcinoma (*n* = 8). Other rare and aggressive types of cancer represented 34 endometrioid cancers (Table 2).

For the cases with endometrial cancer, unique malignant features were found in most of the cases (88.23%, *n* = 135), for 18 other cases the malignant features were associated with cervicitis (*n* = 4), endometritis (*n* = 1), ovarian cysts (*n* = 2), endometrial polyps (*n* = 4), cervical dysplasia (*n* = 1), and leiomyoma (*n* = 2). No significant statistical associations were found between endometrial cancer and adenomyosis or endometriosis, or endometrial polyps. 

### 3.2. IETA Ultrasound Criteria

The echogenity of the endometrium presents significant statistical differences for different situations. In benign pathology, it has a homogeneous profile, while in endometrial cancer and atypical hyperplasia its echogenity is inhomogeneous. A complete inhomogeneous ultrasound aspect is typical for EC (110 of the 111 cases presented this feature), while the presence of endometrial cysts can be found in typical or atypical hyperplasia as well as in EC. In the presence of a homogeneous endometrium, EC is rather rare (Table 3). The relative risk for the inhomogeneous aspect of the endometrium in EC compared with atypical EH is 17.228 (95% CI 1.1398 to 260.1928, *p* = 0.0399, NNT 1.480), while in comparison with typical EH it is 113.3701 (95% CI 7.1407 to 1799.9378, *p* = 0.0008, NNT 1.406), therefore in the presence of an inhomogeneous aspect of the endometrium, EC should be suspected. 

The endometrial-myometrial junction presents significant statistical differences for different situations. In benign pathology, it has a regular aspect (55.61%), while in EC and atypical EH it is described as mostly undefined or irregular (99.34%), as it is presented in Table 4. A regular endometrial-myometrial junction was found in only 1% (*n* = 2) of the cases for EC and in 3% (*n* = 4) for atypical EH, while an undefined junction was found in 130 of the 153 cases of cancer (84.97%) and an irregular junction in 21 cases of EC (13.73%), therefore underlining its value as an ultrasound assessment marker. The relative risk for an undefined endometrial-myometrial junction in EC compared with atypical EH is 3.1155 (95% CI 1.1842 to 8.1966, *p* = 0.0213, NNT 1.733). 

The vascular aspect presents differences regarding the diagnosis, as in benign pathology the vessels present flow, but have no single or double subsequent ramification (81.57%), while in EC the dominant vessels (distinct vessels (arterial and/or venous) passing the endometrial junction) present a circular aspect with multiple focal origin vessels or multifocal origin vessels (Table 5). In the presence of an abnormal blood flow or vessels, the relative risk for EC compared with other gynecological pathologies where uterine bleeding is present is 77.9477 (95% CI 110.9498 to 554.8803, *p* < 0.0001, NNT 2.313).

The aspect of intrauterine fluid reveals significant statistical differences with the diagnosis (Table 6). Its presence has a relative risk of 10.2021 (95% CI 7.0793 to 14.7024, *p* < 0.0001, NNT 1.116) for EC compared with other endometrial pathology.

IETA Doppler score for the uterine artery reveals significant differences depending on the pathology (Table 7), the relative risk for EC where the score was 3 or 4 is 2.9335 (95% CI 2.3046 to 3.734, *p* < 0.0001, NNT 1.805) compared with the other endometrial pathology which is associated with uterine bleeding. Odds Ratio for EC when the Doppler score was 3 or 4 is 19.8098 compared with EH (95% CI 10.1737 to 38.5728, *p* < 0.0001). 

**Figure 3:**
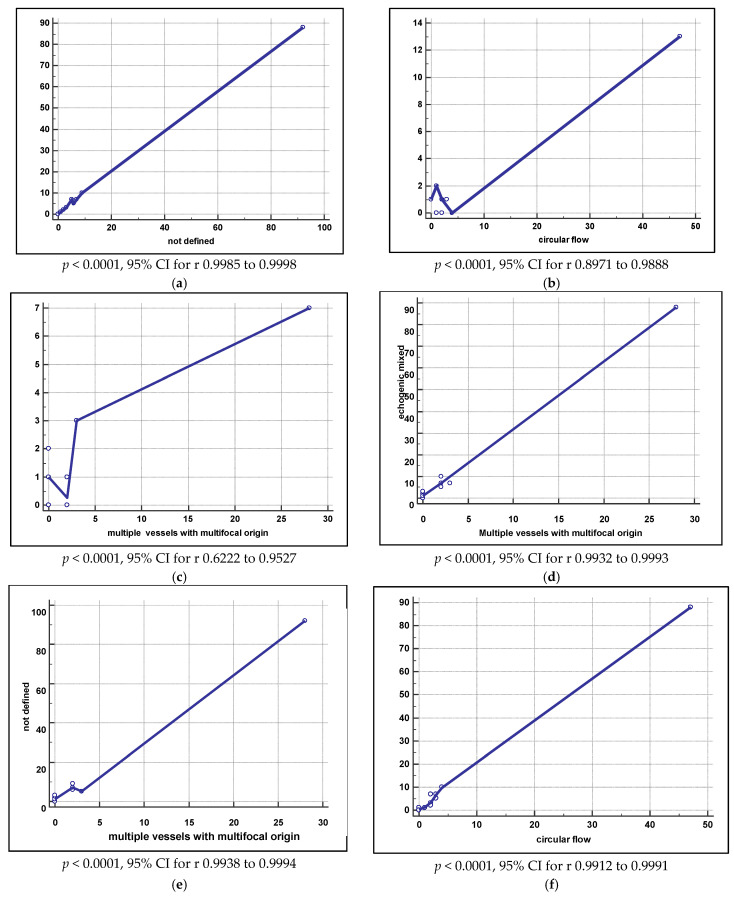
(**a**) Correlation between mixed echogenicity of endometrial fluid and an aspect of endometrial line; (**b**) between ground glass description of endometrial fluid aspects and circular flow; (**c**) non-linear endometrial midline and multiple vessels with multifocal origin vascular features; (**d**) echogenic mixed and multiple vessels with multifocal origin; (**e**) not defined aspects of endometrial midline and multiple vessels with multifocal origin; (**f**) echogenic mixed endometrial fluid with circular flow. Legend: CI—Confidence interval.

Using IETA terminology for describing sonographic features of the 153 EC cases, the results were not defined regarding endometrial midline (*n* = 130, 84.41%), as endometrial fluid was echogenic mixed in 83.66% (*n* = 128), IETA Doppler score reached 4 in 71.90% of cases and vascular features showed multiple vessels with multifocal origin in 24.18% of cases. After analyzing all these parameters for each histopathological aspect, a correlation was found between mixed echogenicity of endometrial fluid and an aspect of endometrial line (Figure 3a–f). The analysis of the EC cases only reveals that EC was of Stage IA in 90.8% of the patients, and 88.9% of tumors were endometrioid (Table 8). 

Sonographic characteristics, according to IETA terminology, of endometrioid and non-endometrioid tumors, with ROC curve analysis and predictive values are presented in Table 9, where in the case of non-endometrioid tumors, the IETA terminology is not validated for any item.

## 4. Discussion

Endometrial cancer and endometrial hyperplasia represent an important public health issue all over the world. Regarding cancer statistics, GLOBOCAN states that in 2018, there were 382,069 new cases of EC and, respectively, 89,929 deaths worldwide [13]. Most sporadic ECs are classified histologically as serous, endometrioid, or clear cell. Each histo-type has a different natural background, a specific clinical appearance/behavior, and a characteristic genetic etiology. In general, ECs endometrioid have a favorable prognosis, being characterized by high frequency genomic changes that influence *ARID1A (BAF250a)*, *CTNNB1 (β-catenin)*, *PIK3CA*, *PIK3R1*, *PTEN*, *KRAS*, and *FGFR2*; additionally, epigenetic silencing of MLH1 is also affected, resulting in microsatellite instability. ECs that are characterized by serous or clear cells are considered clinically aggressive tumors, rare in presentation, but constitute a disproportionate fraction in all deaths caused by EC. Most serous ECs are aneuploid, with frequent genomic changes, affecting *HER-2/ERBB2*, *PPP2R1A*, *PIK3CA,* and *PTEN*, but also *TP53 (p53).* Moreover, they show dysregulation of BAF250a, E-cadherin, cyclin E, and p16. The genetic etiologies of clear cell EC and serous EC are similar, being relatively poorly defined [14]. EEC is considered to be (at presentation) the most common histological subtype. Established epidemiological risk factors for EEC, which inevitably lead to unrestricted estrogenic exposure, include nulliparity, obesity, early menarche, and late menopause, as well as uncontrolled estrogen therapy in postmenopausal women [15]. 

Different clinical and histopathological aspects have been found for EC, but recent systematic reviews have proved the development of endometrioid endometrial cancer and endometrioid adenocarcinoma from hyperplasic lesions in the presence of estrogenic excess; however, the pattern of transformation is rather difficult to predict [16,17,18]. In contrast, a small number of endometrial cancer (especially serous carcinoma) have no connection with estrogenic receptors or its elevated serum levels, as their transformation seems to be from an epithelial atrophy rather than a dysplasia lesion [19]. 

The differences between these two types of EC are present also at a gene level, as microsatellite instability, RAS and PTEN (phosphatase and tensin homolog deleted on chromosome ten) mutations are associated with endometrioid EC and EH, while p53 mutations and abnormal accumulation are associated with serous carcinoma and intraepithelial cancers. Therefore, more complex studies should follow two pathways—an estrogen related one and the alternative non-hormonal one [20]. Based on 366 patients’ observation, Bokhman [21] classified the two major types of EC in type 1—which is dependent on the hormonal misbalance, and type 2—which has no estrogen correlation. 

Atypical endometrial hyperplasia (AH) is without any doubt the premalignant lesion of most, if not all, endometrioid EC type 1 [22]. The similar clinical and ultrasound features between AH and EC type 1 underlines the pathogenic relation between the two lesions. Moreover, histopathological samples reveal their simultaneous presence on close topographic hysterectomy probes and the progression from AH to EC is significant if not treated [23].

Recent studies revealed similar molecular and immunohistochemical markers between AH and EC type 1 [20]. In the absence of data from a larger population screening study, most of the AH cases were diagnosed due to their symptoms, AUB being the most common one. Few prospective studies are available regarding the progression of AH to EC and most of those are limited to surveillance of a patient with a risk factor, such as HRT. Frequently, AH was diagnosed due to post menopause bleeding and not due to an active management. Although using the IETA classification and scoring system can prove helpful in early diagnosis and differentiating benign from malignant features, many cases of EH, especially the AH might need several biopsies due to their unpredictable patterns [24]. 

Although a high number of untreated cases of AH progress to EC and some endometrioid EC can appear without prior AH, AH remains a central premalignant lesion for endometrioid endometrial cancer. EH is commonly found in mixed endometrioid cancers and serous ones rather than in pure serous ones, which suggests that they can start as an endometrioid EC and then develop secondary serous characteristics through an evolution cloning process [25].

Histopathological studies prove furthermore that most serous cancers evolve from a previous distinct lesion, from the intraepithelial endometrial cancer (IEC), which represents the malignant transformation of the atrophic surface of the endometrium [26,27]. The IEC was identified in 89% of the hysterectomy samples with serous type 2 EC and in some of these cases similar lesion to the one of IEC (i.e., EC in situ) were described in the published data [26,27,28]. The most frequent types of EC are the endometrioid adenocarcinoma (EC type 1) which appear in the perimenopause period and are clearly correlated with estrogenic stimulation. They have good prognosis in general for grades 1 and 2 (with good differentiation) and even worse prognosis than EC type 2 for a grade 3. EC type 2 is more common after 60 years of age, in post menopause; it has no correlation with the estrogenic status and is more frequently represented by serous papillary adenocarcinoma and clear cell carcinoma [29]. 

In this study, most of the EC cases were diagnosed in the seventh decade of life (60–69 years) in both counties, but its presence in the fifth decade (40–49 years) is not exceptional, therefore the mean age at diagnosis was 56.41 years. Due to population differences and characteristics depending on geographical regions, any comparison between regions is difficult to assess. However, the standardized rates for EC were evaluated at approximately <40 years in Northern, Western, and Southern Europe, around 45 years in Eastern Europe and around 60 years in the United States of America [30]. Similar, for endometrial hyperplasia, the atypical hyperplasia appears in general over the age of 40 years. Our study reveals a mean age at EH diagnosis of 54 years, which is supported by other studies that appreciate women aged 50–54 are the most affected by this pathology [31]. 

The overall prognosis of EC depends especially on the patient’s age, tumor grading, and depth of invasion and/or cervical involvement and lymph node metastases [31,32,33,34]. 

The value of ultrasonography in nowadays gynecology is undisputed, but its role as a screening tool for asymptomatic patients to intercept an early asymptomatic endometrial cancer might not be efficient from the clinicians’ point of view due to costs. Although we support the use of transvaginal ultrasound in the frame of the routine annual gynecological examination as it increases the chances of an early diagnosis for premalignant and malignant endometrial asymptomatic lesions, as well as for ovarian masses. 

Furthermore, the most relevant study on endometrial thickness measured transvaginal, which included 48,230 postmenopausal women [35], revealed that a cut-off of >5.15 mm is relevant for endometrial hyperplasia and cancer. Due to differences generated by practitioners in measuring and interpretation of ultra-sonographic results [36], a consensual, unique working protocol for ambulatory gynecological assessment is required. IETA (International Endometrial Tumor Analysis group) proposed an algorithm, which includes, besides endometrial thickness, the endometrial volume that might be of help in differentiating benign from malignant endometrial lesions [5,37]. 

Results of the ultrasonography using the IETA algorithm (which was performed for 420 patients (70.70%) of the 594 enrolled in the study) showed that discrimination between EC and EH is possible and feasible. Our research confirmed that inhomogeneous aspect of the endometrium was present in EC and AH versus other endometrial lesions; the endometrial-myometrial junction was regular in benign cases and not in malignant and premalignant ones (where it is irregular or poorly defined); the vascular aspect had no branching in EH (81.57%) compared with multiple vessels with focal or multifocal origin present in EC (this proves once again the role that ultrasound can play in the study of endometrial angiogenesis); the presence of intrauterine fluid was associated with increased EC ratio and therefore its presence should be considered as an alarm sign by clinicians; the characteristics of the intrauterine fluid (where present) had significant differences depending on the diagnosis; and that IETA scores of 3 and 4 highly correlated with the presence of EC. 

Furthermore, strong correlations between the following were confirmed through our study: mixed echogenity of the intrauterine fluid and the irregular aspect of the endometrial-myometrial junction; ground glass intrauterine fluid aspect and the circular endometrial blood flow; mixed ultrasound aspect and multifocal origin of multiple vessels. These correlations and associations can be considered obvious for endometrial cancer and explains the IETA item score of 4 for 72% of the endometrial cancer in our study. Similar correlations and associations were identified for benign lesions as well. IETA scores of 4 were not identified in AH or typical EH and a low IETA score (1) was associated with typical endometrial hyperplasia in 78.94% of the cases, therefore proving once more the efficiency of this algorithm.

## 5. Conclusions

Histopathological examination of endometrial samples remains the standard method for endometrial cancer diagnosis. Routine transvaginal ultrasound assessment of the endometrium in the frame of the routine annual gynecological exam is recommended especially in the perimenopausal period, where it can detect an asymptomatic endometrial hyperplasia or cancer. A consensual, unique working protocol for ambulatory gynecological ultrasound assessment needs to be applied to reduce the differences in the clinical interpretation and management by practitioners. Using the IETA ultrasonography algorithm has proven to be able to distinguish benign from malignant endometrial pathology.

The data obtained in this research are like those of other groups of researchers who used the IETA scoring system. The observed differences can be attributed to the number of cases and the particularities of the population to which it was applied. Additionally, the result obtained promotes the usefulness of introducing the IETA score in current practice, the clinician having additional arguments for the management of abnormal bleeding in perimenopause and menopause

## Figures and Tables

**Table 1:** Demographic data.

Characteristics	Value
Age (years)	56 (13–91)
Age at menopause (years)	49 (32–68)
Body mass index (kg/m^2^)	27.23 (17–38)
Parity	2 (0–9)
0	72 (12.1)
1	104 (17.5)
≥2	418 (70.4)
Use of hormone replacement therapy or local estrogens	11 (1.85)
Postmenopausal	446 (75.1)
Perimenopausal	78 (13.1)
Reproductive age	70 (11.78)

**Table 2:** Number, percentage, and average age for all cases.

Diagnosis	Cases	%	Average Age	SD
EC Type				
Mucinous adenocarcinoma	1	0.65	58.00	-
Pavement-cell carcinoma	1	0.65	85.00	-
Serous endometrioid adenocarcinoma	6	3.92	68.33	6.861
Carcinosarcoma of the uterus	2	1.31	78.50	3.536
Squamous cell carcinoma	4	2.61	68.75	8.302
Endometrioid carcinoma with moderate differentiation	1	0.65	68.00	-
Undifferentiated endometrial carcinoma	2	1.31	59.50	2.121
Mixed cell endometrial carcinoma	3	1.96	60.00	5.568
Endometrial intraepithelial carcinoma (EIC)	2	1.31	56.50	6.364
Adenocarcinoma with squamous differentiation	8	5.23	62.50	9.636
Clear-cell endometrial endometrioid adenocarcinoma	7	4.58	64.00	3.830
Endometrioid adenocarcinoma	101	66.01	63.66	8.998
Villoglandular (papillary) endometrioid adenocarcinoma	10	6.54	64.90	10.290
Endometrioid carcinoma with secretory change	2	1.31	66.00	4.243
Endometrial serous carcinoma	3	1.96	64.67	3.786
**Total**	153	100	64.17	8.794
**Other Lesions**				
Typical EH	133	22.39	49.70	10.279
Atypical EH	14	2.35	58.07	12.413
Adenomyosis/endometriosis	16	2.69	46.60	7.635
Leiomyoma	133	22.39	54.69	10.543
Cervical dysplasia	4	0.67	56.17	11.900
Endometritis	22	3.70	56.91	13.698
Ovarian cyst	10	16.83	51.05	13.683
Cervicitis	13	2.18	58.33	13.172
Endometrial polyps	96	16.16	53.95	11.137

Legend: SD—Std. Deviation, EH—Endometrial hyperplasia.

**Table 3:** Endometrium echogenity in endometrial cancer, endometrial hyperplasia, and other pathologies.

Group *p* < 0.0001	Endometrial Echogenity				Total (no/%)
	Inh	Inh NNRCA	NRCA	Hom	
Other	1/1	39/45	37/56	101/64	178° (42.4)
EC	110/99	21/24	16/24	6/4	153° (36.4)
Atypical EH	0	2/2	3/5	6/4	11° (2.6)
Typical EH	0	24/28	10/15	44/28	78° (18.6)
Total (no/%)	111/26.40	86/20.50	66/15.70	157/37.40	420

Legend: Inh—Inhomogeneous, NNRCA—Inhomogeneous with non-regular cystic areas, NRCA—Inhomogeneous with regular cystic areas, Hom—Homogeneous, EC—Endometrial cancer, EH—Endometrial hyperplasia.

**Table 4:** Endometrial-myometrial junction.

Group *p* < 0.0001	Endometrial-Myometrial Junction			Total (no/%)
	Undefined	Irregular	Regular	
Other	61/27	18/32	99/71	178° (42.4)
EC	130/58	21/37	2/1	153° (36.4)
Atypical EH	3/1,33	4/7	4/3	11° (2.6)
Typical EH	30/13,39	14/25	34/25	78° (18.6)
Total (no/%)	224/53.30	57/13.60	139/33.19	420/100

Legend: EC—Endometrial cancer, EH—Endometrial hyperplasia.

**Table 5:** Color and power Doppler assessment of endometrial vessels.

Group *p* < 0.0001	Vessel Aspect				Total (no/%)
	Dominant Vessels	Without Branching	Focal Origin	Multifocal Origin	
Other	1/1.5	163/64	13/22	1/2.6	178/42.4
EC	67/98.6	14/5.5	35/58	37/97	153/36.4
Atypical EH	0	11/4.3	0	0	11/2.6
Typical EH	0	66/26	12/20	0	78/18.6
Total (no/%)	68/16.20	254/60.50	60/14.30	38/9.00	420/100

Legend: EC—Endometrial cancer, EH—Endometrial hyperplasia.

**Table 6:** Intracavitary fluid in different endometrial pathology.

Group *p* < 0.0001	Intracavitary Fluid			Total
	Mixt Echogenity	Absent	Ground Glass	
Other	73/30 41.0% RT 30.4% CT 17.4% GT	85/73 47.8% RT 72.6% CT 20.2% GT	20/32 11.2% RT 31.7% CT 4.8% GT	178/42.4
EC	128/53 83.7% RT 53.3% CT 30.5% GT	1/1 0.7% RT 0.9% CT 0.2% GT	24/38 15.7% RT 38.1% CT 5.7% GT	153/36.4
Atypical EH	4/2 36.4% RT 1.7% CT 1.0% GT	4/3 36.4% RT 3.4% CT 1.0% GT	3/5 27.3% RT 4.8% CT 0.7% GT	11/2.6
Typical EH	35/15 44.9% RT 14.6% CT 8.3% GT	2723 34.6% RT 23.1% CT 6.4% GT	16/25 20.5% RT 25.4% CT 3.8% GT	78/18.6
Total (no/%)	240/57.1	117/27.9	63/15.0	420/100

Legend: EC—Endometrial cancer, EH—Endometrial hyperplasia.

**Table 7:** IETA Doppler score for the uterine artery.

Group *p* < 0.0001	IETA Doppler Score for the Uterine Artery				Total (no/%)
	1	2	3	4	
Other	120/66	25/49	32/42	1/1	178/42.4
EC	5/3	11/22	27/35	110/99	153/36.4
Atypical EH	6/3	3/6	2/2	0	11/2.6
Typical EH	50/28	12/23	16/21	0	78/18.6
Total (no/%)	181/43.10	51/12.10	77/18.30	111/26.40	420/100

Legend: EC—Endometrial cancer, EH—Endometrial hyperplasia.

**Table 8:** Histological diagnoses of EC.

Characteristics	Values (no/Total (%)
**Stage of cancer for 153 cases**	
IA	139 (90.8)
IC	7 (4.6)
II	7(4.6)
**Histological type**	
Endometrioid	136/153 (88.9)
Grade 1	32/136 (23.5)
Grade 2	83/136 (61.0)
Grade 3	21/136 (15.5)
Non-endometrioid/Grade	17/153 (11.1)
Grade 1	2/17 (11.8)
Grade 2	11/17 (64.7)
Grade 3	4/17 (23.5)
Non-endometrioid/Types	
Serous	5/17 (29.4)
Carcinosarcoma	2/17 (11.8)
Clear-cell carcinoma	4/17 (23.5)
Mixed-cell carcinoma	4/17 (23.5)
Undifferentiated	2/17 (11.8)

**Table 9:** Sonographic characteristics of endometrioid and non-endometrioid tumors.

Ultrasound Feature	AUC (95%CI)	P (Area = 0.5)	SE	SP	PPV° (%) (95%CI)	NPV° (%) (95%CI)	Accuracy
**Endometrioid Tumors (*n* = 136)**							
**Endometrial midline appearance**							
Linear midline	0.73 (0.68–0.77)	<0.0001	98.53	48.24	47.25 (41.28–53.27)	98.58 (94.95–99.83)	64.52
Non-linear midline	0.53 (0.48–0.57)	0.074	90.44	15.49	33.49 (28.65–38.60)	77.49 (64.50–87.48)	39.75
Not defined midline	0.76 (0.72–0.80)	<0.0001	88.97	63.73	53.58 (46.80–60.26)	92.46 (87.85–95.74)	71.90
**Endometrial morphology**							
Heterogeneous with irregular cystic areas	0.55 (0.50–0.60)	0.0056	86.76	23.94	14.56 (8.44–22.77)	92.36 (76.84–98.81)	44.28
Heterogeneous with regular cystic areas	0.57 (0.52–0.62)	<0.0001	94.12	20.42	35.75 (30.75–40.99)	88.06 (77.75–94.73)	44.28
Heterogenous	0.88 (0.84–0.91)	<0.0001	77.94	98.24	95.42 (89.65–98.49)	90.44 (86.61–93.47)	91.66
Homogeneous	0.75 (0.71–0.79)	<0.0001	97.06	53.87	49.75 (43.54–55.96)	97.49 (93.68–99.32)	67.85
**Vascular pattern**							
Multiple vessels with focal origin	0.56 (0.51–0.61)	0.0008	23.53	90.14	97.33 (86.04–99.93)	74.41 (69.73–78.71)	68.57
Multiple vessels with multifocal origin	0.63 (0.58–0.68)	<0.0001	27.21	99.65	97.33 (86.04–99.93)	74.41 (69.73–78.71)	76.19
Without branching	0.91 (0.88–0.93)	<0.0001	95.59	87.32	78.01 (70.90–84.08)	97.67 (94.99–99.15)	89.99
Circular flow	0.71 (0.66–0.75)	<0.0001	44.85	97.54	89.56 (79.69–95.6)	78.98 (74.35–83.11)	80.47
Intrauterine fluid **(IUF)**							
No fluid	0.70 (0.66–0.74)	<0.0001	100	41.2	44.45 (38.77–50.25)	100 (96.91–100)	60.23
“Ground glass”	0.51 (0.47–0.56)	0.3033	87.5	16.2	32.94 (28.09–38.09)	73.36 (60.73–83.71)	39.28
Mixt	0.72 (0.67–0.76)	<0.0001	87.5	57.39	49.14 (42.64–55.66)	90.70 (85.49–94.51)	67.13
IETA DOPPLER score	0.94 (0.91–0.96)	<0.0001	76.47	97.54	52.24 (29.61–74.22)	99.15 (97.68–99.80)	90.71
**Non-endometrioid tumors (*n* = 17)**							
**Endometrial midline appearance**							
Linear midline	0.67 (0.625–0.71)	<0.0001	100	34.49	5.09 (2.83–8.36)	100 (97.39–100)	37.13
Non-linear midline	0.67 (0.62–0.71)	0.0055	47.06	87.84	11.98 (4.84–23.44)	97.92 (95.87–99.12)	86.19
Not defined midline	0.50 (0.45–0.55)	0.9744	47.06	53.35	3.42 (1.35–7.03)	96.62 (93.34–98.57)	53.09
**Endometrial morphology**							
Heterogeneous with irregular cystic areas	0.51 (0.46–0.56)	0.7621	82.35	20.6	3.52 (1.81–6.10)	97.07 (90.95–99.50)	23.09
Heterogeneous with regular cystic areas	0.66(0.61–0.70)	0.0095	47.06	85.61	10.32 (4.15–20.37)	97.86 (95.77–99.10)	84.05
Heterogenous	0.51 (0.46–0.56)	0.7803	76.47	26.55	3.53 (1.77–6.24)	96.97 (91.84–99.29)	28.56
Homogeneous	0.63 (0.58–0.68)	0.0015	88.24	38.46	4.80 (2.55–8.13)	98.93 (95.81–99.90)	40.47
**Vascular pattern**							
Fluid ground glass	0.63 (0.58–0.68)	0.0281	41.18	86.1	9.44 (3.5–19.558)	97.65 (95.49–98.95)	84.28
Multiple vessels with focal origin	0.51 (0.46–0.56)	0.7176	17.65	85.86	4.20 (0.71–12.81)	96.73 (94.33–98.31)	83.10
Multiple vessels with multifocal origin	0.54 (0.49– 0.59)	<0.0001	100	9.43	3.74 (2.074–6.167)	100 (90.808–100)	13.08
Without branching	0.57 (0.52–0.61)	0.2714	52.94	61.04	4.56 (1.934–8.953)	97.35 (94.55–98.95)	60.71
Circular flow	0.6 (0.551– 0.647)	0.0994	35.29	84.62	7.0505 (2.24–15.999)	97.5344 (95.323–98.885)	82.62
Intrauterine fluid **(IUF)**							
Fluid mixt	0.52 (0.47–0.57)	0.7307	47.06	57.32	3.73 (1.48–7.64)	96.85 (93.78–98.67)	56.90
“Ground glass”	0.63 (0.58–0.68)	0.0281	41.18	86.10	9.44(3.5–19.55)	97.65 (95.49–98.95)	84.28
No fluid	0.61 (0.56–0.66)	0.0003	94.12	28.78	4.44 (2.41–7.41)	99.28 (95.58–99.99)	31.41
**IETA DOPPLER score**	0.61 (0.56–0.66)	0.0523	82.35	44.17	4.93(2.55–8.50)	98.61 (95.64–99.76)	45.71

Legend; AUC—Area under curve, EC—Endometrial cancer, P—Significance level, PPV—Positive predictive value, NPV—Negative predictive value, SE—Sensitivity, SP—Specificity.

## Data Availability

All data are available in the archive (data base) of the Emergency Clinical County Hospitals of Arad and Timis, Romania.
